# Supplementary material for: An abundant merozoite surface protein of Plasmodium falciparum modulates susceptibility to inhibitory antibodies
Source: eLife. 2026 Jul 27;14:RP107603. doi: 10.7554/eLife.107603 (PMC13405623; doi:10.7554/eLife.107603)
Supplement: Figure 2—source data 1. — Relevant bands and treatments indicated. [file elife-107603-fig2-data1.zip › Figure 2-source data 1/Figure 2B-Source Data.pdf]

Figure 2B-Source Data: PCR confirmation of 3D7 *msp*2 knock-out. This gel matches to Fig 2B.

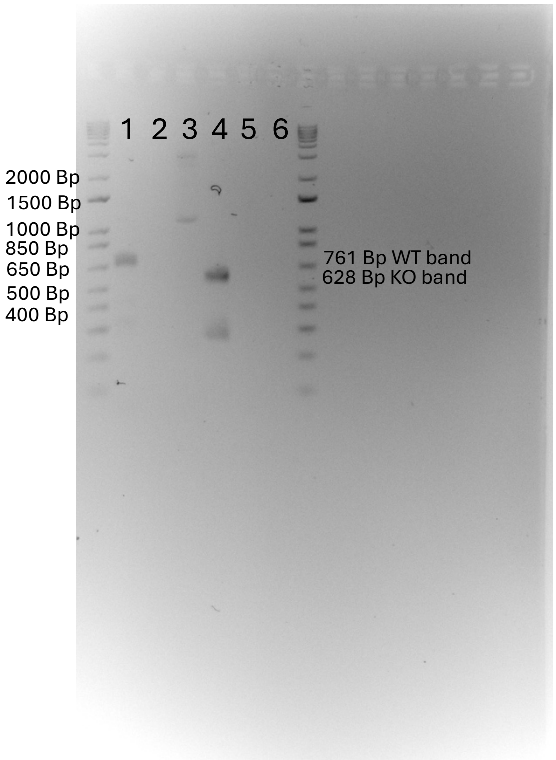

Fig 2B Sample	Sample	Expected Band Size
1 Kb Plus Ladder	N/A	N/A
**1**	**3D7 WT DNA (A+B)**	**761**
**2**	**3D7 WT DNA (A+C)**	N/A
**3**	**3D7 ΔMSP2 Cl1 DNA (A+B)**	N/A
**4**	**3D7 ΔMSP2 Cl1 DNA (A+C)**	**628**
5	Water	N/A
6	Water	N/A
1 Kb Plus Ladder	N/A	N/A

Samples in Bold used in Figure 2B.
N/A= not applicable
