# Supplementary material for: An abundant merozoite surface protein of Plasmodium falciparum modulates susceptibility to inhibitory antibodies
Source: eLife. 2026 Jul 27;14:RP107603. doi: 10.7554/eLife.107603 (PMC13405623; doi:10.7554/eLife.107603)
Supplement: Figure 2—source data 1. — Relevant bands and treatments indicated. [file elife-107603-fig2-data1.zip › Figure 2-source data 1/Figure 2C-Source Data.pdf]

Figure 2C-Source Data: Western Blot Confirmation of 3D7 MSP2 knock-out in schizonts in comparison to aldolase and GAP45 (schizont expressed). These gels match to Fig 2C.

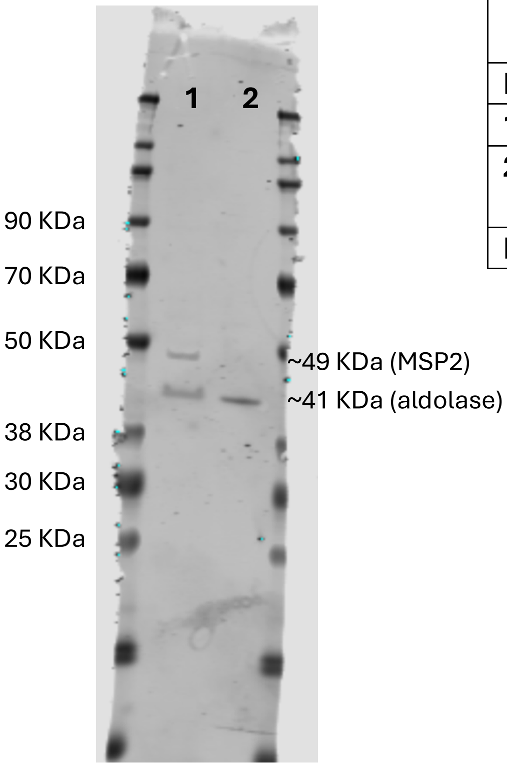

Fig 2C (MSP2/aldolase) Sample	Sample	Expected Band Size
Ladder (Chameleon)	N/A	N/A
**1**	**3D7 WT**	**~49 kDa**
**2**	**3D7 ΔMSP2 Cl1**	Absent
Ladder (Chameleon)	N/A	N/A

Primary antibodies:
Anti-MSP2 11E1 (mouse mAb)
Anti-aldolase (rabbit)

Secondary antibodies:
IRDye 800CW goat anti-mouse
IRDye 680RD goat anti-rabbit

Samples presented in Figure 2 (MSP2/aldolase) are indicated in Bold.
N/A= not applicable

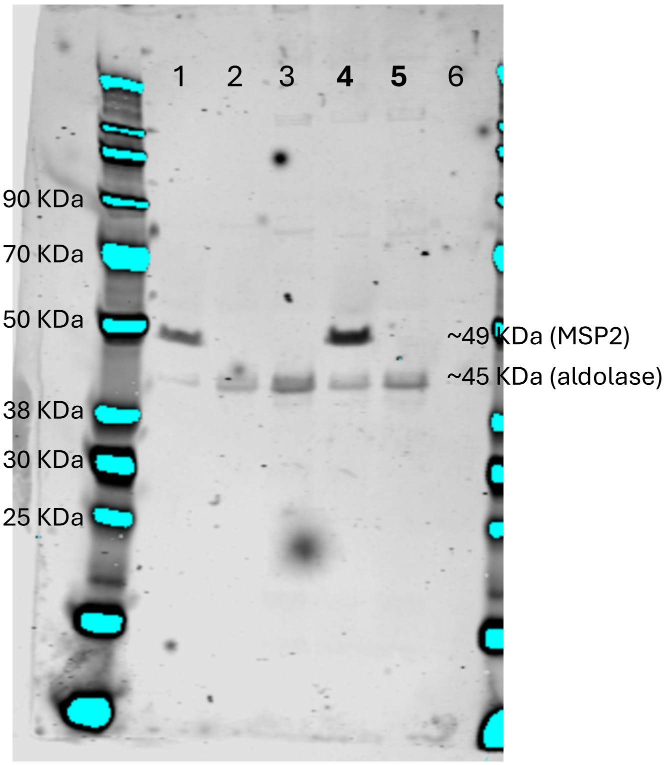

Fig 2C (MSP2/GAP45) Sample	Sample	Expected Band Size
Ladder (Chameleon)	N/A	N/A
1	3D7 WT	~49 kDa
2	3D7 ΔMSP2 Cl1	~45 kDa
3	3D7 ΔMSP2 Cl2	~45 kDa
**4**	**3D7 WT**	**~49 kDa**
**5**	**3D7 ΔMSP2 Cl1**	**~45 kDa**
6	3D7 ΔMSP2 Cl2	~45 kDa
Ladder (Chameleon)	N/A	N/A

Primary antibodies:
Anti-MSP2 2F2 (mouse mAb)
Anti-GAP45 (rabbit)

Secondary antibodies:
IRDye 800CW goat anti-mouse
IRDye 680RD goat anti-rabbit

Samples presented in Figure 2 (GAP45) are indicated in Bold.
N/A= not applicable
