# Supplementary figures and images for: An abundant merozoite surface protein of Plasmodium falciparum modulates susceptibility to inhibitory antibodies
Source: eLife. 2026 Jul 27;14:RP107603. doi: 10.7554/eLife.107603 (PMC13405623; doi:10.7554/eLife.107603)

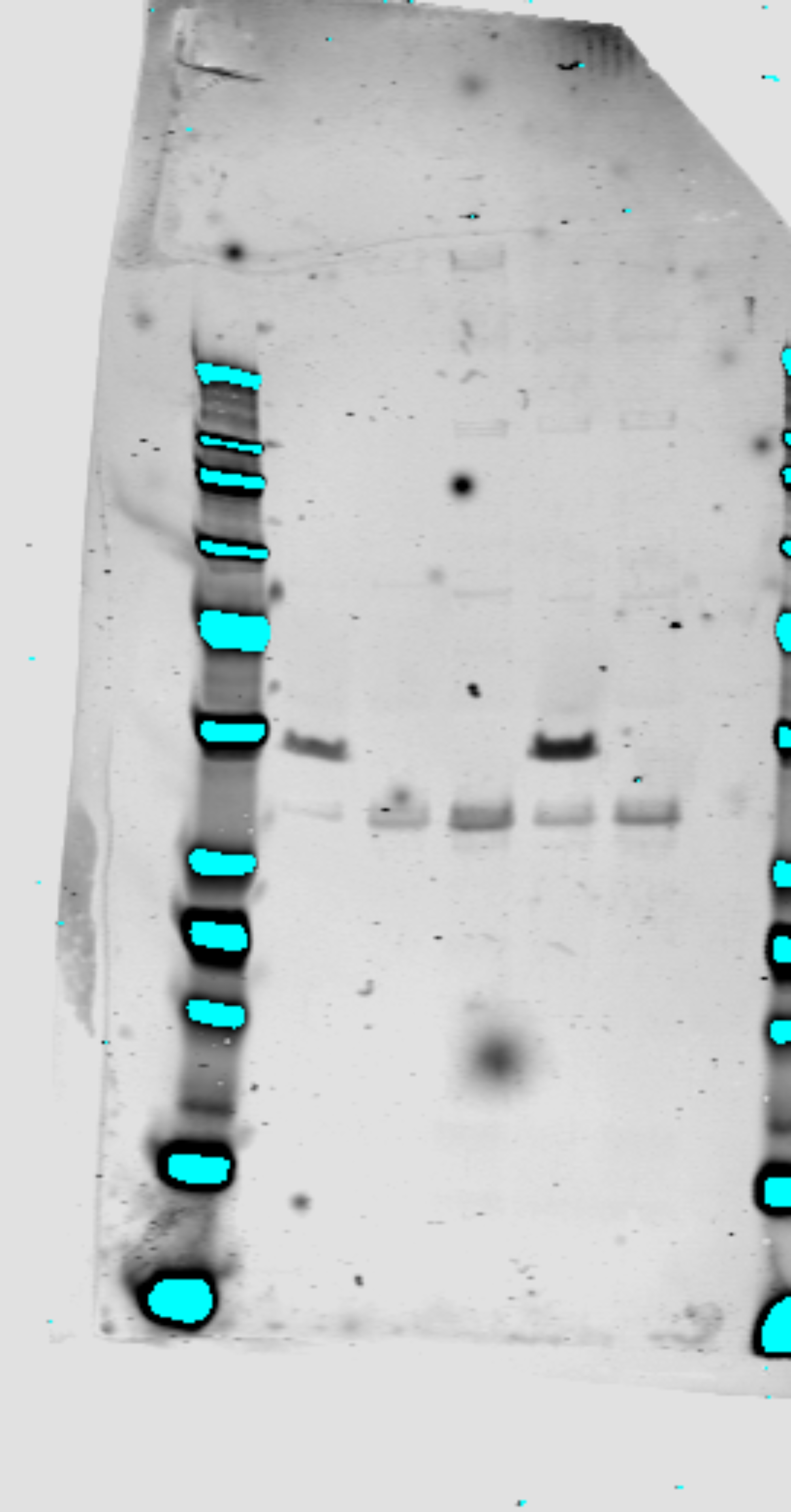

Supplement: Figure 2—source data 2. [file elife-107603-fig2-data2.zip › Figure 2-source data 2/Figure 2C-Source Data anti-GAP45 Raw Image.png]

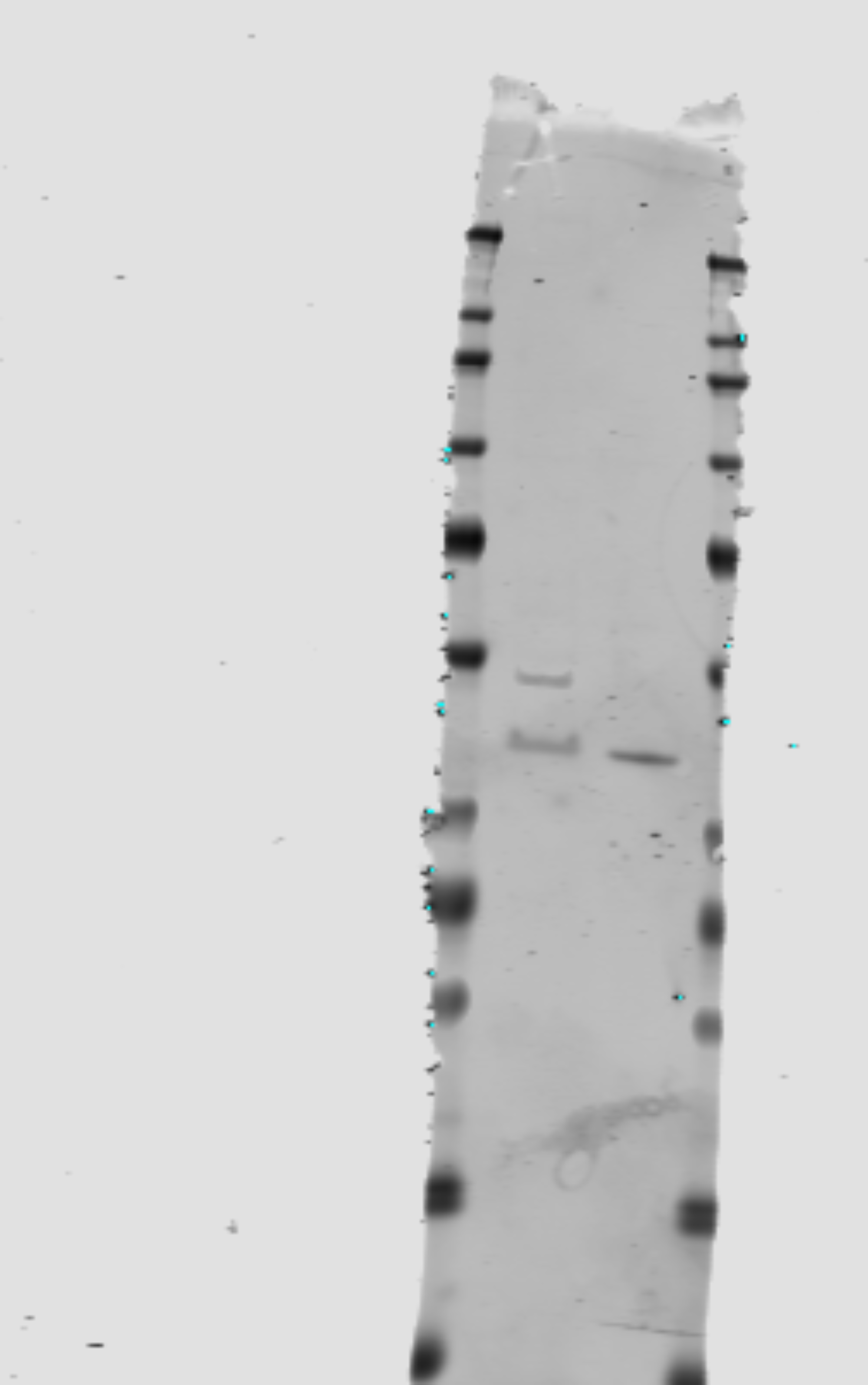

Supplement: Figure 2—source data 2. [file elife-107603-fig2-data2.zip › Figure 2-source data 2/Figure 2C-Source Data anti-Aldolase Raw Image.png]

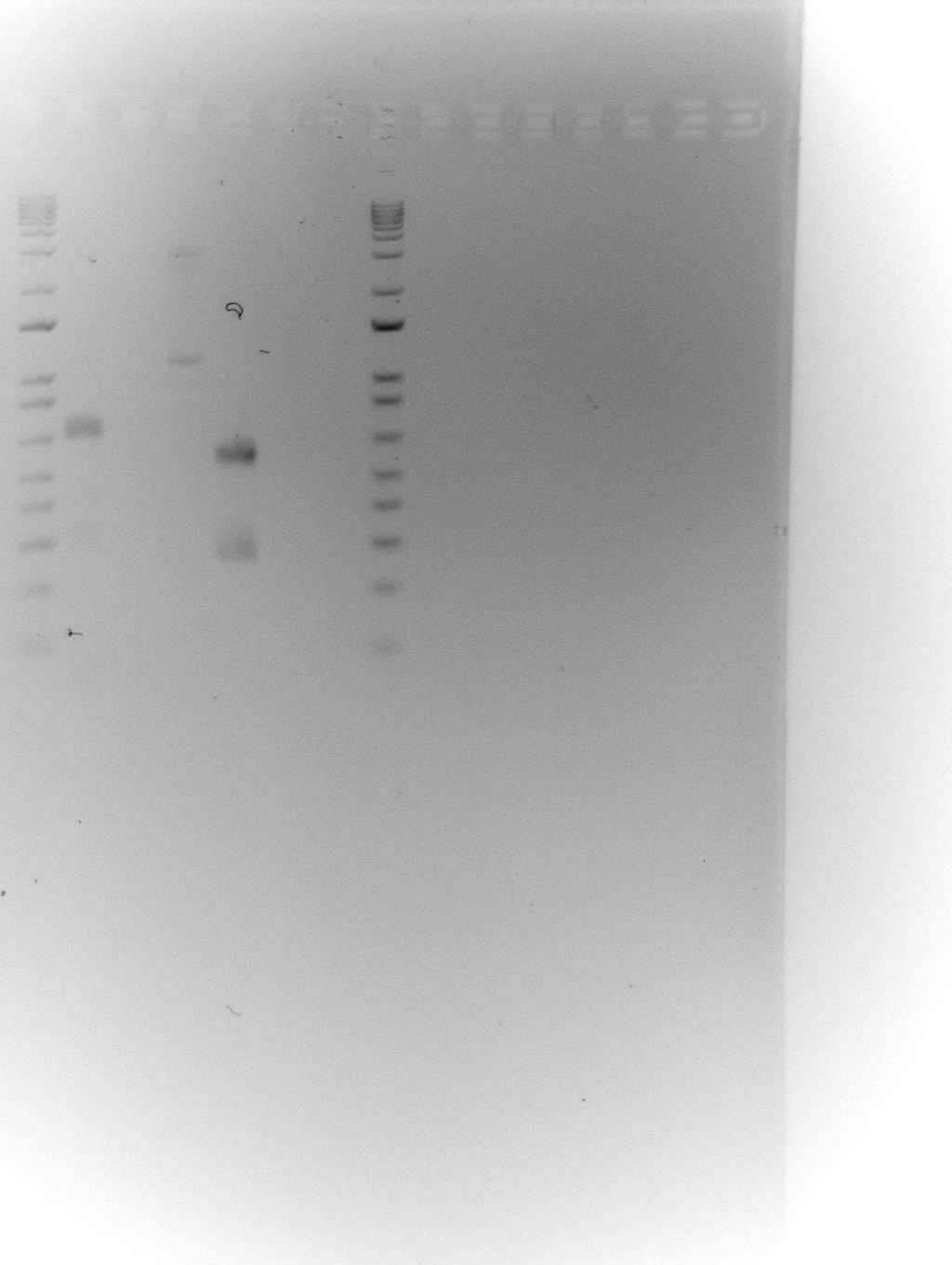

Supplement: Figure 2—source data 2. [file elife-107603-fig2-data2.zip › Figure 2-source data 2/Figure 2B-Source Data Raw Image.png]

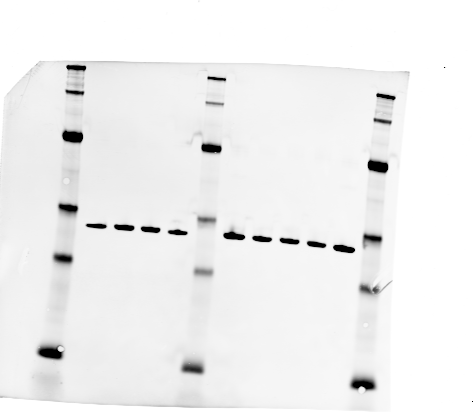

Supplement: Figure 3—source data 2. [file elife-107603-fig3-data2.zip › Figure 3-source data 2/Figure 3C-Source Data anti-EXP2 Raw Image.png]

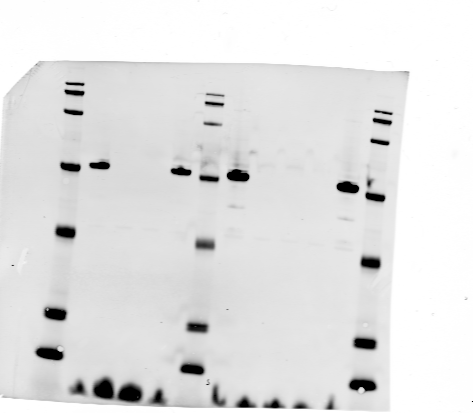

Supplement: Figure 3—source data 2. [file elife-107603-fig3-data2.zip › Figure 3-source data 2/Figure 3C-Source Data anti-MSP2 Raw Image.png]

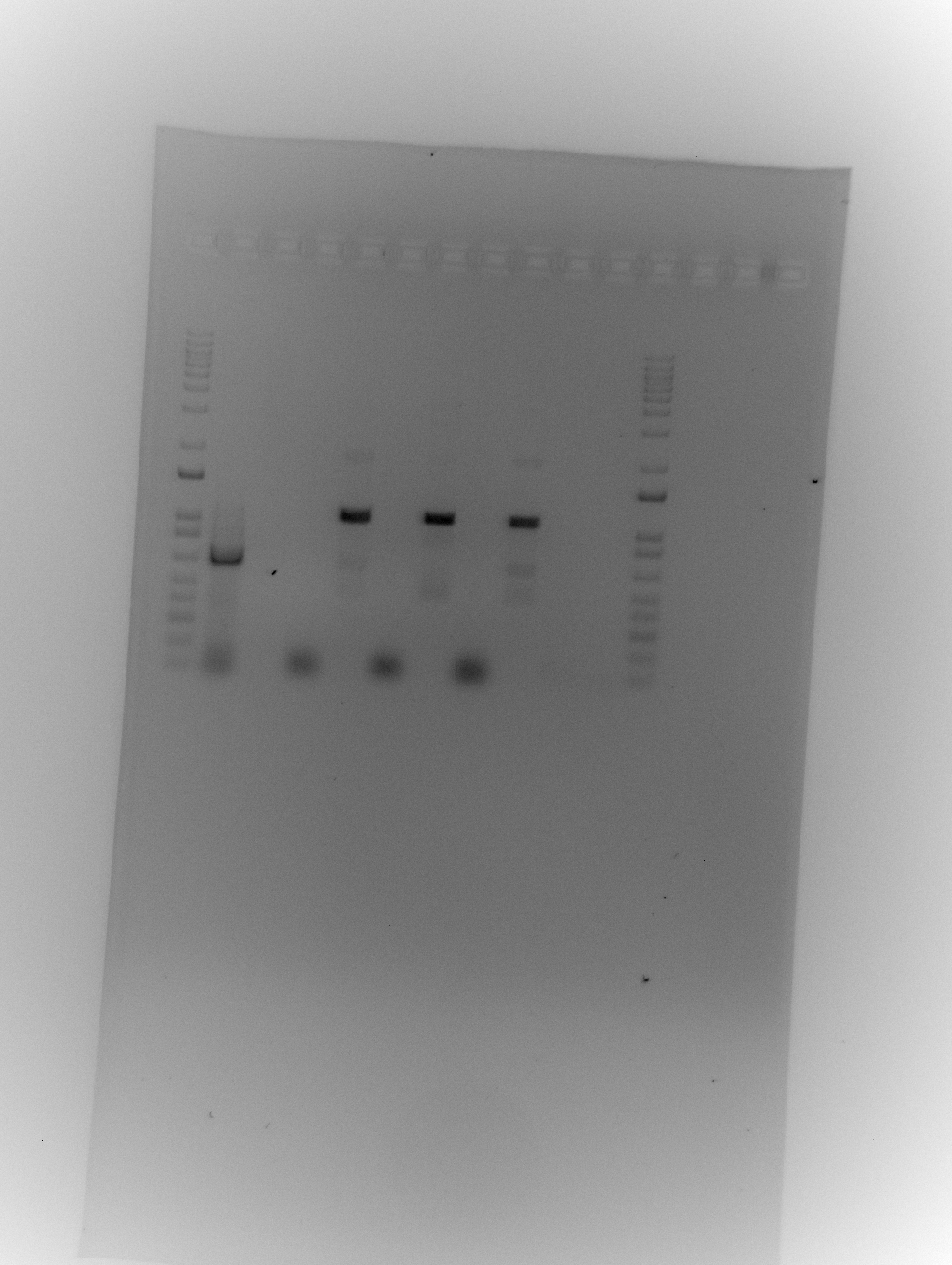

Supplement: Figure 3—source data 2. [file elife-107603-fig3-data2.zip › Figure 3-source data 2/Figure 3B-Source Data Raw Image.png]
