# Supplementary material for: An abundant merozoite surface protein of Plasmodium falciparum modulates susceptibility to inhibitory antibodies
Source: eLife. 2026 Jul 27;14:RP107603. doi: 10.7554/eLife.107603 (PMC13405623; doi:10.7554/eLife.107603)
Supplement: Figure 3—source data 1. — Relevant bands and treatments indicated. [file elife-107603-fig3-data1.zip › Figure 3-source data 1/Figure 3C-Source Data.pdf]

Figure 3C-Source Data: Western Blot Confirmation of Dd2 MSP2 knock-out in schizonts in comparison to loading control EXP2. This gel matches to Fig 3C.

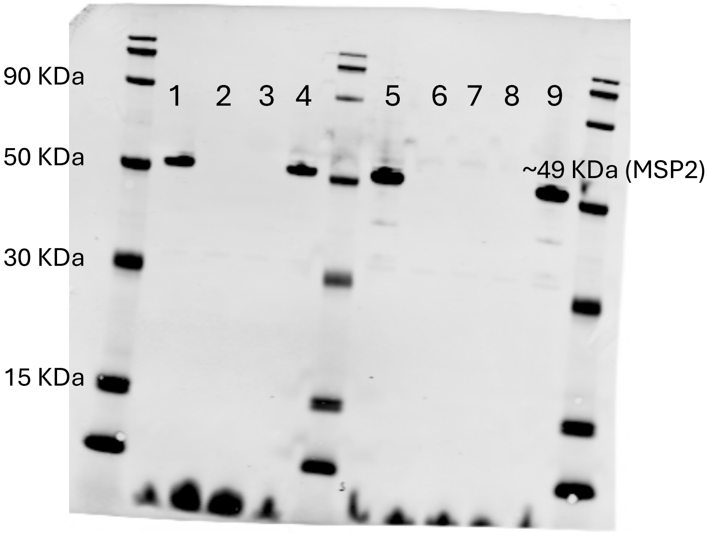

Fig 3C (MSP2) Sample	Sample	Expected Band Size
Ladder (Chameleon)	N/A	N/A
**1**	**Dd2 WT**	**~49 kDa**
**2**	**Dd2 ΔMSP2 Cl1**	**Absent**
3	Dd2 ΔMSP2 Cl2	Absent
4	Dd2 WT	~49 kDa
Ladder (Chameleon)		
5	Dd2 WT	~49 kDa
6	Dd2 ΔMSP2 Cl1	Absent
7	Dd2 ΔMSP2 Cl2	Absent
8	Dd2 ΔMSP2 Cl3	Absent
9	Dd2 WT	~49 kDa
Ladder (Chameleon)	N/A	N/A

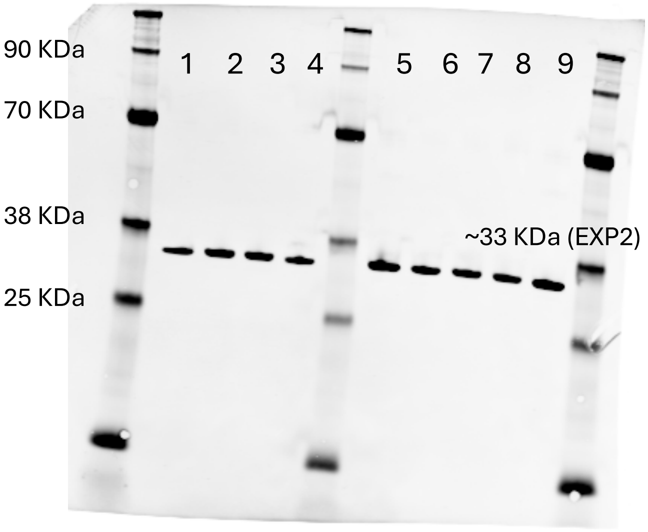

Fig 3C (EXP2) Sample	Sample	Expected Band Size
Ladder (Chameleon)	N/A	N/A
**1**	**Dd2 WT**	**~33 kDa**
**2**	**Dd2 ΔMSP2 Cl1**	**~33 kDa**
3	Dd2 ΔMSP2 Cl2	~33 kDa
4	Dd2 WT	~33 kDa
Ladder (Chameleon)	N/A	N/A
5	Dd2 WT	~33 kDa
6	Dd2 ΔMSP2 Cl1	~33 kDa
7	Dd2 ΔMSP2 Cl2	~33 kDa
8	Dd2 ΔMSP2 Cl3	~33 kDa
9	Dd2 WT	~33 kDa
Ladder (Chameleon)	N/A	N/A

Primary antibodies:
Anti-FC27 MSP2 (rabbit)
Anti-EXP2 (mouse mAb)

Secondary antibodies:
IRDye 800CW goat anti-mouse
IRDye 680RD goat anti-rabbit

Samples presented in Figure 3C are indicated in Bold.
Samples 1-4 represent harvest 1, samples 5-9 represent harvest 2.
N/A= not applicable
