# Supplementary material for: An abundant merozoite surface protein of Plasmodium falciparum modulates susceptibility to inhibitory antibodies
Source: eLife. 2026 Jul 27;14:RP107603. doi: 10.7554/eLife.107603 (PMC13405623; doi:10.7554/eLife.107603)
Supplement: Figure 3—source data 1. — Relevant bands and treatments indicated. [file elife-107603-fig3-data1.zip › Figure 3-source data 1/Figure 3B-Source Data.pdf]

Figure 3B-Source Data: PCR confirmation of Dd2 *msp*2 knock-out. This gel matches to Fig 3B.

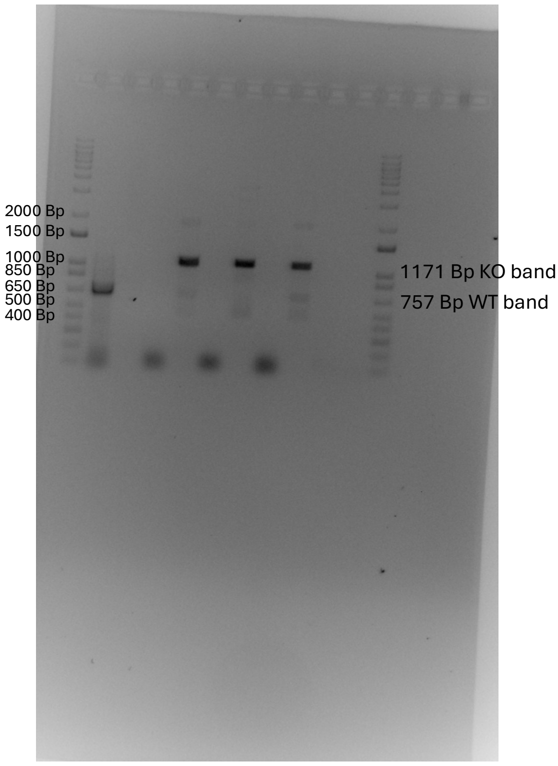

**Fig 3B Sample**	**Sample**	**Expected Band Size**
1 Kb Plus Ladder	N/A	N/A
**1**	**Dd2 WT DNA (A+B)**	**757**
**2**	**Dd2 WT DNA (D+E)**	N/A
**3**	**Dd2 ΔMSP2 Cl1 DNA (A+B)**	N/A
**4**	**Dd2 ΔMSP2 Cl1 DNA (D+E)**	**1171**
5	Dd2 ΔMSP2 Cl2 DNA (A+B)	N/A
6	Dd2 ΔMSP2 Cl2 DNA (D+E)	**1171**
7	Dd2 ΔMSP2 Cl3 DNA (A+B)	N/A
8	Dd2 ΔMSP2 Cl3 DNA (D+E)	**1171**
9	Water	N/A
10	Water	N/A
1 Kb Plus Ladder	N/A	N/A

Samples in Bold used in Figure 3B.
N/A= not applicable
