# Supplementary material for: An abundant merozoite surface protein of Plasmodium falciparum modulates susceptibility to inhibitory antibodies
Source: eLife. 2026 Jul 27;14:RP107603. doi: 10.7554/eLife.107603 (PMC13405623; doi:10.7554/eLife.107603)
Supplement: Supplementary file 1. [file elife-107603-supp1.pdf]

Supplementary File 1. Primers used to generate MSP2 KO lines.

5' flank MSP2 KO F	GGTCCGCGGCTCGACTAATCAATTTACAATTTC	Sac11
5' flank MSP2 KO R	GGTACTAGTGCCATACTTCTCCTTATACTC	Spe1
3' flank MSP2 KO F	GGTGAATTCCTCTTCATTTTAAAACATTGAC	Ecor1
3' flank MSP2 KO R	GGTCCATGGGTTTTTTCAATGCGTGC	Nco1
Dd2 MSP2 KO 5' flank F	GGTCCGCGGCTCGACTAATCAATTTACAATTTCC	Sac11
Dd2 MSP2 KO 5' flank R	GGTACTAGTCTAGTAGTATTAGAACCTTCATTTG	Spe1
Dd2 MSP2 KO 3' flank F	GGTGAATTCATTCTCTTCATTTTAAAACATTGAC	EcoR1
Dd2 MSP2 KO 3' flank R	GGTCCATGGGTACTTGAAGAAATATGGTACC	Nco1
PfMSP2 guide 1 F	TAAGTATATAATATTTGGTAATGGTGCAGATGCTGGTTTTAGAGCTAGAA	
PfMSP2 guide 1 R	TTCTAGCTCTAAAACCAGCATCTGCACCATTACCAAATATTATATACTTA	
Dd2 MSP2 Guide 2 F	TAAGTATATAATATTGCACCAGAGAATAAAGGTACGTTTTAGAGCTAGAA	
Dd2 MSP2 Guide 2 R	TTCTAGCTCTAAAACGTACCTTTATTCTCTGGTGCAATATTATATACTTA	
5' int 3D7 MSP2 KO F	CTTTTCATATAATAAATCAAATGCAC	
5' int 3D7 MSP2 KO R	TACAAAATGCTTAAGACAGATC	
3D7 WT 5' int check R	GGTAGTTGTGGTAGTAGC	
Dd2 5' check F	CAATTACGATATAAAACCTAGTATCTTTC	
Dd2 WT 5' int check R	CTATTTGTACTCCTTTGACTTCCAC	
