# Supplementary material for: An abundant merozoite surface protein of Plasmodium falciparum modulates susceptibility to inhibitory antibodies
Source: eLife. 2026 Jul 27;14:RP107603. doi: 10.7554/eLife.107603 (PMC13405623; doi:10.7554/eLife.107603)
Supplement: Supplementary file 2. [file elife-107603-supp2.pdf]

Supplementary File 2. Antibodies used in this study.

**Serum/Antibody/ibody**	**Source**
Anti-MSP2 mAb 2F2	Adda et al Infect Immun 2012
Anti-MSP2 Rb FC27	Robin Anders, LaTrobe University
Anti-EXP2 Rb	Bullen et al. J Biol Chem 2012
Anti-PfAldolase	Abcam
Anti-PfRh2b Rb 1055-3	Lopaticki et al Infect and Immun 2011
Anti-PfEBA175/Rh2b Rb 1056-3	Lopaticki et al Infect and Immun 2011
Anti-PfEBA175/Rh4 Rb 1049-3	Lopaticki et al Infect and Immun 2011
Anti-PfEBA175/Rh2b/Rh4 Rb 1067-3	Lopaticki et al Infect and Immun 2011
Anti-w2mef EBA175 Rb	Healer et al PLoS One 2013
Anti-MSP1-19 Rb (6858, 645, 647)	Paul Gilson, Burnet Institute
Anti-Rh5.1 Rt 494	Healer et al Front Cell Infect Microbiol 2022
Anti-PTRAMP nAb H8	Scally et al Nat Microbiol 2022
Anti-CSS nAb D2	Scally et al Nat Microbiol 2022
Anti-CSS nAb D2-FC	Scally et al Nat Microbiol 2022
Anti-AMA1 3D7 Rb (1072, 1149, 1161)	Drew et al. PLoS One 2012
Anti-AMA1 w2mef Rb (1151, 1164, 1163, 128/7)	Drew et al. PLoS One 2012
Anti-AMA1 mAb 1F9	Coley et al PEDS 2001
Anti-AMA1 mAb 4G2	Collins et al J. Biol. Chem. 2007
Anti-AMA1 ibody WD33	Angange et al Nat Comms 2025
Anti-AMA1 ibody WD34	Angange et al Nat Comms 2025
Anti-AMA1 WD34	Angange et al Nat Comms 2025
Anti-AMA1 ibody WD34-FC	Angange et al Nat Comms 2025

Rb= rabbit. mAb= mouse monoclonal. Rt= rat. nAb= camelid nanobody. I-body= single domain antibody derived from shark variable new antigen receptor (V_{NAR}).
