# Supplementary material for: An abundant merozoite surface protein of Plasmodium falciparum modulates susceptibility to inhibitory antibodies
Source: eLife. 2026 Jul 27;14:RP107603. doi: 10.7554/eLife.107603 (PMC13405623; doi:10.7554/eLife.107603)
Supplement: Supplementary file 3. [file elife-107603-supp3.pdf]

Supplementary File 3. Enzymes used to remove/inactivate RBC receptors and target residues of the enzyme.

**Enzyme**	**Cleavage Target**	**Supplier**
Neuraminidase (0.067 U/mL)	Sialic acid residues	Sigma-Aldrich
High Trypsin (1 mg/mL)	Lysine or Arginine residues, unless followed by a proline	Sigma-Aldrich
Low Trypsin (0.067 mg/mL)	Lysine or Arginine residues, unless followed by a proline	Sigma-Aldrich
Chymotrypsin (1 mg/mL)	Tyrosine, Tryptophan or Phenylalanine	Worthington Biochemical Corporation
Chymotrypsin (1 mg/mL)/ Trypsin (0.067 mg/mL)	Tyrosine, Tryptophan, Phenylalanine, Lysine or Arginine residues, unless followed by a proline	Worthington Biochemical Corporation & Sigma-Aldrich
