# Supplementary material for: An abundant merozoite surface protein of Plasmodium falciparum modulates susceptibility to inhibitory antibodies
Source: eLife. 2026 Jul 27;14:RP107603. doi: 10.7554/eLife.107603 (PMC13405623; doi:10.7554/eLife.107603)
Supplement: Supplementary file 4. [file elife-107603-supp4.pdf]

Supplementary File 4. Primers used for RT- qPCR of *P. falciparum* MSP2, MSP5, MSP4 and controls.

Name	Sequence
FrucBiAld 3D7 F qPCR [a, b]	TGTACCACCAGCCTTACCAG
FrucBiAld 3D7 R qPCR [a,b]	TTCCTTGCCATGTGTTCAAT
MSP2 3D7 F qPCR	TCCTACTGCACAACCTGAACAA
MSP2 3D7 R qPCR	ATGTCCATGTTGTCCTGTACCTT
MSP4 3D7 F qPCR	CAAAAGAATCCCAAATGGTTGATGATAA
MSP4 3D7 R qPCR	AACATGGCCACCTGAATTTGAT
MSP5 3D7 F qPCR	AAATTAATGAGAATGCAGAAATAGGTCAA
MSP5 3D7 R qPCR	CGCTATAATGTGGCACCTCAT
SUB1 3D7 F qPCR[c]	GGAATGAGGTAGATGCCGATGAA
SUB1 3D7 F qPCR[c]	TCCTTTACATCTTTCAGTTCCCTCAT

[a] FrucBisAld primers had been previously validated as an appropriate housekeeping gene by Salanti et. al. (2003). Primers listed here are the fructose-bisphosphate aldolase qPCR primers (primer pair 61) from Salanti et. al. (2003).

[b] fructose-bisphosphate aldolase is ubiquitously expressed throughout the parasite lifecycle and is used as a non-stage specific housekeeping gene.

[c] SUB1 is a schizont expressed protein and is used as a schizont-stage specific housekeeping gene.
