# Supplementary material for: An abundant merozoite surface protein of Plasmodium falciparum modulates susceptibility to inhibitory antibodies
Source: eLife. 2026 Jul 27;14:RP107603. doi: 10.7554/eLife.107603 (PMC13405623; doi:10.7554/eLife.107603)
Supplement: Peer review history

# Editor report: eLife Assessment

**DOI:** 10.7554/eLife.107603.4.sa0

**Authors:** Urszula Krzych

**Affiliations:** Walter Reed Army Institute of Research United States

**Keywords:** Compelling, Important

This **important** work challenges current models of merozoite surface protein function by showing that MSP2 is dispensable for parasite growth while modulating immune responses to AMA1, with implications for malaria vaccine design. The conclusions are supported by **compelling** experimental evidence, including state-of-the-art technologies and well-characterized monoclonal antibodies. These findings provide new insights into immune evasion and antigen targeting that will be of broad interest to parasitology, immunology, and vaccine researchers.

# Referee report: Joint Public Review

**DOI:** 10.7554/eLife.107603.4.sa1

[Editors' note: this version has been assessed by the Reviewing Editor without further input from the original reviewers.]

The major strengths of the manuscript are in the *Plasmodium falciparum* genetic and phenotyping approaches. PfMSP2 knockouts are made in two different strains, which is important as it is know that invasion pathways can vary between strains, but is a level of comprehensiveness that is not always delivered in *P. falciparum* genetic studies. The knockout strains are characterised very thoroughly using multiple different assays and the authors should be commended for publishing a good deal of negative data, where no phenotype was detected. This is not always done but is very helpful for the field and reduces the potential for experimental redundancy, i.e., others repeating work that has already been performed but never published. The quality of the writing, referencing and figures is also generally strong.

There are certainly some areas of the manuscript that would benefit from deeper exploration, such as electron microscopy/other imaging approaches to explore whether deletion of PfMSP2 has a visible impact on merozoite surface structure, further replicates of the video microscopy assays to see whether trends in the data could reach significance (although these are very time-consuming and technically difficult assays), and follow up of some of the genes where expression is changed by PfMSP2 knockout (as the authors point out, there are no candidates that have a very obvious link to invasion suggesting that they may be compensating for PfMSP2 function, although several are expressed in schizont stages). However, there is already a substantial amount of data in the manuscript, and more detailed follow-up is reasonable to leave to future work. Overall, with the modifications made through the review process, including the addition of new controls for key experiments, the claims and conclusions are justified by the data, and the manuscript generates important new information about a highly studied *Plasmodium falciparum* merozoite surface protein. The studies are important and have potential for directing vaccine design targeting erythrocyte invasion, a critical step in bloodstream expansion of malaria parasites.

# Author comment: Author response

**DOI:** 10.7554/eLife.107603.4.sa2

**Authors:** Isabelle G Henshall, Jill Chmielewski, Dimuthu Angange, Ornella Romeo, Keng Heng Lai, Kaitlin R Turland, Nicki Badii, Michael Foley, Robin F Anders, James Beeson, Danny W Wilson

**Affiliations:** The University of Adelaide Adelaide Australia; The University of Adelaide Adelaide Australia; La Trobe University Melbourne Australia; The University of Adelaide Adelaide Australia; The University of Adelaide Adelaide Australia; The University of Adelaide Adelaide Australia; La Trobe University Melbourne Australia; Adalta Pty Ltd Melbourne Australia; La Trobe University Melbourne Australia; Burnet Institute Melbourne Australia; The University of Adelaide Adelaide Australia

The following is the authors’ response to the previous reviews

**Public Reviews:**

**Reviewer #2 (Public review):**

(1) There are certainly some areas of the manuscript that would benefit from deeper exploration, such as electron microscopy/other imaging approaches to explore whether deletion of PfMSP2 has a visible impact on merozoite surface structure.

We in principle agree with the reviewer that applying enhanced resolution microscopy approaches to understand structural and functional changes with loss of *Pf*MSP2 could be of interest. However, based on our ongoing work, this represents a significant body of work in terms of experimental optimisation in an effort to gain the detail required to make meaningful insights. Therefore, this will remain outside the scope of this manuscript and we hope to provide these insights in future studies.

(2) Further replicates of the video microscopy assays to see whether trends in the data could reach significance (although these are very time-consuming and technically difficult assays).

Conclusions we have drawn from live-cell imaging data for MSP2 knock-out parasites encompass some 43 invading merozoites from 21 schizont ruptures for *Pf*Dd2 WT and 35 invading merozoites from 18 schizont ruptures for *Pf*Dd2 DMSP2 parasites. One of the leading studies to apply live-cell microscopy to film invading merozoites based conclusions of invasion kinetics on: 3D7 (number of merozoite invasion = 63, number of schizont ruptures = 23), D10 (invasions = 33, ruptures = 20) and W2mef (invasions = 39, ruptures = 15; this line is of the same lineage as Dd2) (Weiss et al. PLoS Pathogens, 2015). Although there are variations within and between lines from this gold-standard study, our dataset is mostly comparable in terms of the number of schizont ruptures and merozoite invasions filmed and analysed to look at changes in kinetics. What we can say definitively is that there is no strong phenotype in the absence of inhibitory antibodies against other antigens for either live-cell or growth inhibition assays. Therefore, we have focussed the data interpretation in the manuscript to highlight the lack of statistical significance and limited phenotype seen, which given the previously believed importance of MSP2 to *P. falciparum* invasion of red blood cells is somewhat surprising.

In order to address this suggestion, we have modified the discussion to better represent any non-significant changes in invasion and growth seen.

“Despite the abundance of *Pf*MSP2 on the merozoite surface and previous work suggesting a role in RBC invasion, we found merozoites invade and grow with similar kinetics to wildtype parasites in the absence of *Pf*MSP2. This does not exclude a role for *Pf*MSP2 in vivo where there are additional pressures, such as immune-effector mechanisms and flow dynamics, on merozoite invasion. However, given we have knocked-out *Pf*MSP2 from two different *P. falciparum* isolates, our findings do not currently support a major role for *Pf*MSP2 in the mechanics of merozoite invasion. Thus, it appears that the function of the two most abundant proteins on the merozoite surface, *Pf*MSP1 (Das et al., 2015; Kals et al., 2024) and *Pf*MSP2, are not obviously linked to merozoite binding to the RBC and subsequent invasion.”

(3) Follow up of some of the genes where expression is changed by PfMSP2 knockout (as the authors point out, there are no candidates that have a very obvious link to invasion suggesting that they may be compensating for PfMSP2 function, although several are expressed in schizont stages).

A thorough investigation of the genes where expression changes with PfMSP2 knock-out would require a substantial body of additional work, not least because they would all have to be investigated as there is no single likely candidate based on stage of expression, membrane binding properties or previous links to merozoite surface architecture. Given this, potential follow up of these proteins will be left for future studies.

We also thank the reviewer for the recognition of the work provided in the manuscript and the modifications made that have improved the manuscript from version 1. The reviewer also recognises the value in our detailed characterisation, including data where phenotyping changes with MSP2 knock-out could not be seen, in defining the function of *Pf*MSP2 as commented below:

However, there is already a substantial amount of data in the manuscript, and more detailed follow-up is reasonable to leave to future work. Overall, with the modifications made through the review process, including the addition of new controls for key experiments, the claims and conclusions are justified by the data, and the manuscript generates important new information about a highly studied *Plasmodium falciparum* merozoite surface protein.

**Reviewer #3 (Public review):**

Major points:

(1) Much of the manuscript describes negative results and this reviewer found it arduous to get through many negative or nonsignificant results before finally getting to the significant effect on AMA1 inhibitory antibodies, not presented until Figure 6! Computational studies in Fig. 1 could be a supplementary figure. Figs. 2 and 3. demonstrate knockout in 3D7 and Dd2, respectively and could be assembled into a single figure. (Notably Fig. 2A and 3A are almost identical with use of some different primers.) Fig. 2E, 2F, 3D-H, all of Fig. 4, most of Fig. 5 are all negative or insignificant results that could also be moved to supplementary data. As MSP4, MSP5, and SUB1 are presumably included in the whole genome RNA-seq experiments shown in Fig. 4C, it makes sense to remove Fig. 4A data from the paper fully. These consolidating changes would help highlight the key finding of improved binding and block of AMA1's role in invasion.

We have chosen to not take the approach proposed by Reviewer 3 as it would leave the manuscript with only around 2.5 Figure panels and undersells the very significant amount of work that has been done to characterise *Pf*MSP2 knock-out lines. Although, as noted by the reviewer, piggyBac mutagenesis studies predict *Pf*MSP2 is dispensable, much of the field likely expect *Pf*MSP2 to be essential to *P. falciparum* blood stage parasite growth due to the results of earlier reverse genetics approaches and many years of publications that have speculated on the importance of the protein. Therefore, we are also conscious of providing very clear and comprehensive evidence to support our findings. While this may delay highlighting the findings in Figure 6, we also note that the lengths we have gone to in characterising an important antigen with a difficult phenotype is still valued as evidenced by Reviewer 2 (Public Review Comments on the original manuscript):

“PfMSP2 knockouts are made in two different strains, which is important as it is known that invasion pathways can vary between strains, but is a level of comprehensiveness that is not always delivered in *P. falciparum* genetic studies. The knockout strains are characterised very thoroughly using multiple different assays, and the authors should be commended for publishing a good deal of negative data, where no phenotype was detected.”

(2) The potentiating effects on anti-AMA1 antibodies are shown with rabbit sera and purified antibodies, mouse monoclonal antibodies, and smaller i-bodies inspired by shark antibody-like receptors but not with human monoclonal antibodies (hmAbs). As naturally acquired hmAbs targeting AMA1 have been identified and characterized (PMIDs: 39632799, 40020675), would it not be important to test these antibodies in the ∆MSP2, especially as the authors emphasize the importance of their model in designing better human malaria vaccines?

As the reviewer noted, we demonstrated enhanced inhibitory activities of antibodies to AMA1 using rabbit polyclonal antibodies, mouse mAbs, and i-bodies. We note that the WD34 i-Body we used was humanised to be IgG-like with a human Fc-region (IgG1 backbone). Rabbit IgG is very similar to human IgG1. Therefore, we have provided evidence of the enhancing effect using different types and sources of antibodies relevant to human immunity to support our conclusions. Our findings open new avenues for future research and we agree with the reviewer that future studies using panels of human mAbs to defined epitopes would be interesting and may further inform vaccine design; however this is beyond the scope of the current paper. We do not have the mAb mentioned by the reviewer to test in our system. To perform studies with human mAbs would take a substantial amount of time (many months), requiring the generation of different human mAbs and quantification of their activity and testing them for potentiation effects. While this would be an interesting future endeavour, we do not feel that such studies are needed at this stage to support our conclusions, and instead would be a future extension from our current paper. To acknowledge the reviewer's comment, we have extended our comment in the discussion about future studies with different panels of invasion inhibitory antibodies to include huMabs targeting AMA1 as follows:

“Further investigation using the parasite lines developed in this study and a wider panel of antibodies that target different stages of the merozoite invasion process, including human monoclonal antibodies against AMA1 (Patel et al., 2025), could shed more light on this potentially novel mechanism of vaccine derived antibody efficacy.”

(3) Fig. 7 presents quantitative fluorescence microscopy to measure anti-AMA1 binding and support a model where MSP2 serves to sterically hinder antibody access to AMA1 on individual merozoites. I understand that the negative WD33 control is useful to contrast to the positive WD34 antibody (both bind AMA1 but only WD34 exhibits parasite growth inhibitory effects), but it seems that use of smaller i-bodies rather than conventional larger mouse or ideally human monoclonal antibodies may compromise demonstration of steric hindrance by MSP2 because smaller i-bodies may be less hinder.

The antibodies used in this experiment have fluorescent tags attached. So while the untagged WD33 and WD34 i-bodies are approximately 14 kDa, when fused to GFP or mCherry their expected size increases to approximately 42 kDa, approaching that of the Fc-tagged WD34 i-body (78 kDa) that shows increased growth inhibitory activity in the absence of MSP2. Therefore, we expect steric hindrance to be a significant factor with these fluorescently tagged antibodies.

(4) Some explanation for why WD33 fails to inhibit growth despite targeting the same antigen as WD34 is needed. Are the epitopes known? Does one bind further from the RON2 binding pocket?

As reported in Angage et al., Nature Communications 15, 7206 (2024). WD34 has been identified to bind to, and block, a site within the hydrophobic AMA1 and RON2 binding pocket found on Domain II of AMA1. In contrast, WD33 recognises a distinct conserved epitope in Domain II of AMA1 near to, but not overlapping with, the hydrophobic AMA1 and RON2 binding pocket. We have clarified this by including additional description when first describing the i-bodies as follows:

“When we tested the i-body WD34 (Angage et al., 2024) which binds a highly conserved epitope that includes the *Pf*RON2-binding pocket on *Pf*AMA1 domain II, we observed a small potentiation of *Pf*AMA1 specific activity with knock-out of *Pf*MSP2 in *Pf*3D7 (1.3-fold; IC_50_ *Pf*D7 WT 0.012 mg/mL; IC_50_ *Pf*3D7 DMSP2 0.009 mg/mL; p=0.08 Figure 6F).”

Then

“A second i-body, WD33 (Angage et al., 2024), which binds AMA1 between domain II and domain III but does not appear to overlap with the *Pf*RON2-binding pocket on *Pf*AMA1, had very limited invasion inhibitory activity against *Pf*3D7 parasites and did not show improved potency with knock-out of *Pf*3D7 MSP2 (0.9-fold; IC_50_ *Pf*3D7 WT 1.02 mg/mL; IC_50_ *Pf*3D7 DMSP2 1.1 mg/mL; p=0.8; Figure 6I).”

**Recommendations for the authors:**

**Reviewing Editor Recommendations:**

Although providing microscopic images might require a lengthy process, including results based on human mAbs (if available) might enhance the strength of evidence. The reorganization of the figures and the presentation of results usually falls into the realm of personal preferences, however, if the comments/suggestions are useful, it might highlight your message.

As covered in the Response to Public Reviewer Comments for Reviewer 2 and indicated by the editor, investigations of phenotypes found in this study using high-resolution imaging techniques (e.g. electron microscopy) will require very significant additional work and will be attempted in future studies. We also provide a response to Reviewer 3 in regards to the potential to test human monoclonal antibodies and believe this is best done more thoroughly in future studies. We have elected to not make substantial changes to the data presented as suggested by Reviewer 3. We have addressed additional comments as covered below.

**Reviewer #3 (Recommendations for the authors):**

Minor Comments

(1) Scale bar in Fig. 7A is not resolved well. The image is too pixelated to resolve merozoites or the actual dimensions of the scale bar.

We have updated this figure to provide improved clarity of the scale bar.

(2) Lines 69, 216, 221, 253, 628-629, 648 all suggest that MSP2 was heretofore assumed to be essential. However, piggyBac insertional mutagenesis revealed that MSP2 is highly dispensable (MIS of 0.988, per PlasmoDb.org; PMID: 29724925). I would suggest to tone down this claim as it does not detract from the authors' production of useful ∆MSP2 clones.

We agree with the reviewer that the piggyBac insertional mutagenesis study results should also be acknowledged and apologise for this oversight. To address this, we have reviewed the sentences highlighted by the reviewer and, where appropriate for the historical interpretation of *Pf*MSP2 function, have added the following modified information through the text:

“*P. falciparum* merozoite surface protein 2 (*Pf*MSP2), an antigen reported to be refractory to gene knock-out in *P. falciparum* (Sanders et al., 2006) but that has also been reported to be dispensable in a piggyBac mutagenesis study (Zhang et al., 2018), has been of long-term interest as a vaccine candidate.”

“Given previous unsuccessful attempts to disrupt *pfmsp2* (Sanders et al., 2006), and its high abundance on the merozoite surface (Gilson et al., 2006), *Pf*MSP2 has been traditionally viewed as an essential *P. falciparum* protein with an essential function in merozoite invasion, although more recent piggyBac mutagenesis studies have called this understanding into question (Zhang et al., 2018).”

We have chosen not to modify this text and it remains the same as below. The reason for not changing this text is the result that we could knock-out MSP2 from 3D7 was still unexpected given the published reverse genetics studies and results from piggyBac mutagenesis studies are also sometimes not reliable indicators of what happens when reverse genetics is performed. Therefore, the following text we believe is a reasonable description.

“Unexpectedly, we confirmed successful disruption of *pfmsp2* by replacing the coding sequence between 132 bp and 819 bp of the gene with a hDHFR drug selection cassette in the 3D7 *P. falciparum* laboratory-adapted line (Figure 2A and B), resulting in *Pf*3D7 DMSP2 parasites.”

“As a previous reverse genetics study in 3D7 reported that *Pf*MSP2 was essential for *P. falciparum* growth in vitro (Sanders et al., 2006), we investigated whether *Pf*MSP2 could also be removed from *Pf*Dd2, an isolate of *P. falciparum* that differs from 3D7 in geographical origin, RBC receptor usage and allelic type of *pfmsp2*.”

“However, CRISPR-Cas9 gene editing used in this work has shown that, in contrast to previous attempts to knock-out *Pf*MSP2 (Sanders et al., 2006), *Pf*MSP2 is not essential for *P. falciparum* blood stage parasite growth in vitro.”

“Advancements in gene-editing techniques in *P. falciparum* have allowed us to directly demonstrate using reverse genetics in two different parasite lines that *Pf*MSP2 is not essential for *P. falciparum* growth in vitro.”

(3) Figs. 2B, 2C, 2D show PCR, immunoblots, and IFA with a ∆MSP2 clone but two clones (termed clone 1 and clone 2) are show in panels 2E and 2F. Which clone is used in each panel? Without clarification, readers may wonder if one clone was used for PCR but another clone gave a desired result in immunoblots? By convention, validation studies (PCR and immunoblots) should be performed and shown (in Supplementary figures) for all clones used for phenotype studies; alternatively, a single clone can be used throughout if all clones are presumed identical. Which of these clones was used for the RNA-seq experiments in Fig. 4C? Similar questions arise for the two knockout clones made in the Dd2 line (Fig. 3D).

We agree with the reviewer that it would be helpful to have this information provided more clearly through the Results. To this end, we have updated the Figure legends across Figures 2, 3, 4, 5, 6, 7 and Supplementary Figure 5 as appropriate to specifically indicate the clones used for the downstream experiments. All clones were validated by PCR and, after growth characteristics were found to be the same, a single clone was used for all downstream experiments for *Pf*MSP2 knock-outs in both 3D7 and Dd2.
